# Patient-derived organoids in gastric cancer: bridging the tumor microenvironment to functional precision oncology

**DOI:** 10.3389/fbioe.2026.1790902

**Published:** 2026-05-22

**Authors:** Ji Ma, Xinyang Liu, Hongtao Xu, Chuan Jiang

**Affiliations:** 1 Gastrointestinal Surgery Department, Lishui Municipal Central Hospital, The Fifth Affiliated Hospital of Wenzhou Medical University, Lishui, China; 2 Department of Dermatology, Lishui Municipal Central Hospital, The Fifth Affiliated Hospital of Wenzhou Medical University, Lishui, China

**Keywords:** gastric cancer, immune evasion, patient-derived organoids, precision oncology, tumor microenvironment

## Abstract

Gastric cancer (GC) represents a formidable global health challenge characterized by profound molecular heterogeneity and a dynamically evolving tumor microenvironment (TME). Although genomic sequencing facilitates precision medicine, its capacity to capture intricate tumor microenvironment interactions or predict individual therapeutic outcomes remains limited. Recently, patient-derived cancer organoids (PDCOs) have emerged as high-fidelity three-dimensional (3D) models that faithfully recapitulate the histological architecture, mutational landscapes, and functional phenotypes of their parent tumors. This review systematically delineates advances in using GC-PDCOs to simulate the TME and elucidate immune evasion mechanisms, specifically camouflage, coercion, and cytoprotection. We emphasize the transformative potential of PDCOs as “patient avatars” in functional precision oncology, highlighting their promising predictive value for sensitivities to chemotherapy, targeted therapies, and immunotherapies, with early small-cohort studies demonstrating high clinical concordance. Furthermore, we evaluate the integration of emerging technologies, such as artificial intelligence and 3D bioprinting, to overcome current translational bottlenecks. Finally, we propose a strategic roadmap for integrating organoid technology into clinical workflows to achieve truly individualized therapeutic regimens for every gastric cancer patient.

## 1 Introduction

Gastric cancer (GC) remains a formidable global health challenge, ranking as the fifth most frequently diagnosed malignancy and a leading cause of cancer-related mortality worldwide (Kan et al., 2025). According to 2022 statistics, GC accounted for approximately 970,000 new cases and 660,000 deaths annually, highlighting its status as a primary driver of the global cancer burden (Bray et al., 2024). Despite advancements in screening and multi-modality treatments, the clinical prognosis remains suboptimal, primarily due to the subtle nature of early symptoms leading to frequent late-stage diagnoses when curative options are limited (Migliore et al., 2025). The disease is characterized by pronounced inter-patient molecular variability and high intra-tumoral heterogeneity, which frequently results in divergent responses to standardized therapeutic regimens (Pectasides et al., 2018; Ma et al., 2025). While the integration of targeted agents, such as those against HER2, VEGF, and Claudin 18.2, and immune checkpoint inhibitors (ICIs) like anti-PD-1 antibodies has expanded the therapeutic landscape, only a subset of patients derives significant clinical benefit, emphasizing a critical clinical bottleneck in overcoming treatment resistance and tailoring interventions to individual tumor biology (Shitara et al., 2023).

The shift toward personalized oncology has traditionally relied on genomic profiling to identify actionable mutations; however, next-generation sequencing often falls short of predicting functional therapeutic outcomes because it cannot fully replicate the complex, dynamically evolving *in vivo* microenvironment (Zhu et al., 2021). Functional precision medicine has emerged to address this gap, necessitating preclinical models that faithfully mirror a patient’s unique cancer profile. Patient-derived cancer organoids (PDCOs) have revolutionized this field as three-dimensional (3D) *in vitro* systems that can be rapidly established from surgical resections or biopsies (Xiang et al., 2024). Unlike conventional two-dimensional (2D) cell lines, which often lose key tumor features during prolonged passaging, PDCOs exhibit strong self-organizing properties and accurately recapitulate the histological structure, genetic stability, and mutational landscapes of their parent tumors (Polak et al., 2024). These “mini-tumors” provide a scalable and physiologically relevant platform for *ex-vivo* drug testing, enabling the prospective assessment of chemotherapy, radiotherapy, and targeted therapies within a clinically actionable timeframe (Wang et al., 2025a). While the financial resources required for organoid culture are moderate compared to 2D cell lines, their superior predictive value significantly reduces the costs associated with ineffective treatments and toxicity management (Ferreira et al., 2025). Furthermore, the 7–14-day establishment cycle from endoscopic biopsies aligns seamlessly with routine clinical staging and surgical planning, facilitating the practical integration of high-throughput organoid biobanks into standard hospital workflows (Zhao et al., 2026).

This review systematically examines the transformative potential of gastric cancer organoids (GC-PDCOs) in bridging the gap between laboratory research and clinical decision-making. We discuss recent technological milestones that allow GC-PDCOs to function as “patient avatars,” facilitating individualized drug screening and the identification of potential biomarkers for treatment sensitivity and resistance (Du et al., 2025). A central focus is placed on the integration of advanced co-culture systems, including air-liquid interface (ALI) and microfluidic organ-on-chip platforms, which enable the reconstruction of the native tumor microenvironment (TME) by incorporating autologous immune cells and stromal components (Zhao et al., 2024). By authentically replicating tumor-immune interactions, these models offer unprecedented insights into the mechanisms of immune evasion and the role of the intratumoral microbiota in GC progression (Wang et al., 2025a). Finally, we highlight the challenges of clinical translation, such as the need for standardized protocols and rigorous validation through multi-institutional trials, while envisioning a future where GC-PDCOs serve as an integral component of a multi-omic, artificial intelligence (AI)-driven precision oncology pipeline (Wang et al., 2025a; Zhu et al., 2025).

## 2 GC-PDCOs as high-fidelity disease models

### 2.1 Biological advantages and consistency

GC-PDCOs serve as high-fidelity disease models by maintaining the intrinsic biological features of the parent tumor across multiple dimensions (Skubleny et al., 2025; Yin et al., 2025). Histologically, PDCOs preserve the specific glandular architecture, protein expression profiles, and nuclear stratification characteristic of Lauren subtypes, including intestinal, diffuse, and mixed patterns (Zhao et al., 2024; Skubleny et al., 2025). This morphological fidelity is robustly supported by the stable expression of key protein markers such as MUC5AC, CK7, and CEA, which exhibit high concordance with the original patient tissues in nearly 95% of cases (Xu et al., 2024a; Yoon et al., 2023). Beyond morphology, the genomic landscape, comprising single-nucleotide variants, copy number variations, and transcriptomic profiles, remains remarkably stable during *ex vivo* cultivation. Large-scale biobanking studies have demonstrated that PDCOs accurately reflect the mutational profiles of critical gastric cancer driver genes, such as TP53, ARID1A, and CDH1, maintaining over 95% of common mutations found in the coding sequences of matched primary tumors (Zu et al., 2023; Wang et al., 2014). This genetic stability ensures that PDCOs represent a reliable surrogate for studying the evolutionary dynamics and molecular stratification of individual patients.

**FIGURE 1:**
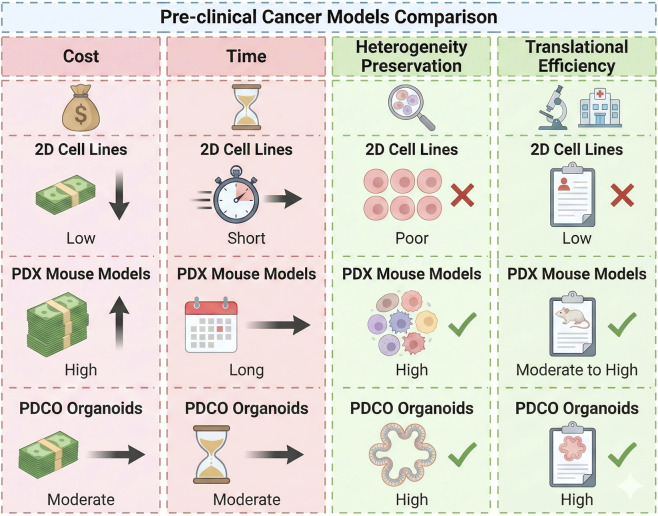
Comparative analysis of preclinical models for gastric cancer research.

The functional utility of GC-PDCOs in precision oncology is primarily anchored in their ability to accurately replicate the pharmacological response and drug resistance traits of the donor patient (Kim et al., 2025a; di Paola et al., 2025). Preliminary clinical correlation studies have reported promising concordance between *ex-vivo* drug sensitivity testing and actual patient outcomes, particularly for standard-of-care regimens like FLOT, FOLFOX, and targeted agents, although large-scale validations are still required (Yoon et al., 2023). Compared to traditional 2D cell lines, which fail to imitate the three-dimensional architecture and lack the phenotypic and genetic heterogeneity of human tumors, PDCOs provide a far more complex and physiologically relevant framework for disease modeling (Kastner et al., 2021). Furthermore, while patient-derived xenografts (PDXs) remain a significant tool for *in vivo* studies, they are frequently limited by long establishment cycles (up to 4–8 months), low engraftment efficiency, high maintenance costs, and the progressive replacement of human stromal cells with murine counterparts during passaging (Zhang et al., 2025). In contrast, GC-PDCOs can be established from minimal tissue amounts, such as endoscopic biopsies, within 24 h and utilized for high-throughput drug screening within a clinically actionable timeframe of approximately 2 weeks (McDonald et al., 2023). This rapid turnaround time and superior scalability make PDCOs an indispensable platform for prospective clinical decision-making and functional precision oncology (Figure 1).

### 2.2 Capturing heterogeneity

The defining clinical challenge of GC lies in its profound intratumoral heterogeneity (ITH) and inter-lesional variability, which frequently drive therapeutic resistance and disease progression (Pisică-Ilinca et al., 2025; Kim et al., 2025b). PDCOs have demonstrated a remarkable capacity to preserve this multi-dimensional heterogeneity, offering a unique platform for high-resolution molecular dissection. Unlike traditional models, PDCOs can be established as multiple clonal lines from spatially distinct regions of a single tumor or from various metastatic sites, such as the liver, ovaries, or peritoneum (Joo et al., 2025). Recent single-nuclei and single-cell RNA sequencing (scRNA-seq) analyses have confirmed that these organoid lines effectively replicate the genetically diverse subpopulations found in the original tissue (Foo et al., 2022; Yang and Yu, 2023). For instance, comparative scRNA-seq studies between primary tumors and matched organoids reveal that while *in vitro* culture may deplete certain immune-cell populations, such as lymphoid and plasma cells, the epithelial signatures and cancer-intrinsic transcriptional programs remain highly conserved (Kumar et al., 2022).

Furthermore, scRNA-seq has been instrumental in validating the role of GC-PDCOs as a bridge between Lauren’s histological classification and modern molecular stratification. Trajectory analysis of organoid single-cell transcriptomes has revealed a state of “transcriptional plasticity”, where tumor-associated epithelial cells undergo pervasive and ongoing differentiation and dedifferentiation (Wang et al., 2021). This lineage fluidity is particularly evident in studies of gastric intestinal metaplasia (GIM) organoids, where clonal derivatives from a single progenitor can give rise to a continuous spectrum of cells ranging from hybrid gastric-intestinal states to advanced intestinal differentiation (Yue et al., 2025). These “hybrid” states are characterized by the reactivation of fetal gene programs and YAP-dependent regenerative signatures, mimicking the high-altitude lineage plasticity often observed in malignant transformation (Yue et al., 2025).

Crucially, PDCO models allow researchers to look beyond pure histology to identify clinically relevant genetic phenotypes that may be obscured in bulk sequencing. Recent studies have identified specific intestinal-type organoid clones that, despite their glandular histology, exhibit high CD44 expression and diffuse-like genetic characteristics associated with one-carbon (1C) metabolic reprogramming (Lõhmussaar et al., 2021; Wensink et al., 2021). This finding highlights a positive correlation between stemness marker intensity and 1C metabolism enrichment, suggesting that targeting the CD44-1C metabolism axis could provide a precision therapeutic strategy for a subpopulation of genetically diffuse-like gastric cancers (Zhao et al., 2023a). By integrating chromosomal aberrations, such as chromosome 20 gain, with transcriptomic profiles, these organoid platforms can model the earliest stages of neoplastic evolution and identify the high-risk subpopulations predisposed to anchorage-independent growth and metastatic spread (Yue et al., 2025; Heinz et al., 2022). To mitigate the risk of sampling bias, a multi-site sampling strategy—involving biopsies from spatially distinct primary regions and multiple metastatic sites—is essential to capture the full spectrum of a patient’s genetic diversity and ensure the high-fidelity representation of the disease state (Chen et al., 2026a).

### 2.3 Key technological milestones

The evolution of GC-PDCOs is defined by a series of pivotal breakthroughs that transitioned the technology from foundational stem cell biology to its current status as a cornerstone of functional precision oncology (Tang et al., 2025). The journey began with the landmark identification of Lgr5 as a definitive marker for epithelial stem cells within the gastric units (Fatehullah et al., 2021). The subsequent development of culture systems capable of sustaining Lgr5^+^ stem cells *in vitro* allowed for the generation of long-lived, self-organizing gastric units that faithfully recapitulated the architecture of the native epithelium (Barker et al., 2010). This “bottom-up” approach provided the necessary blueprint for transforming primary patient tissues into 3D models, establishing the feasibility of maintaining the genetic and phenotypic integrity of the human stomach *ex-vivo* (Shao et al., 2025).

Building upon this foundation, the field shifted toward the systematic establishment of large-scale patient-derived organoid biobanks (Shao et al., 2025; Liu et al., 2025a). These repositories have proven instrumental in capturing the profound molecular heterogeneity of gastric cancer, representing a diverse array of subtypes including chromosomal instability (CIN), microsatellite instability (MSI), and genomically stable (GS) tumors (Fatehullah et al., 2021). A critical milestone in this era was the demonstration that GC-PDCOs could serve as “patient avatars” for high-throughput drug screening. Pilot studies have shown strong potential in predicting clinical responses to standard chemotherapies, such as the FLOT regimen, and targeted agents (Schmäche et al., 2024). Furthermore, the integration of orthotopic transplantation models facilitated the study of tumor-resident stem-cell-like pools in a physiological context, confirming that these populations are essential not only for tumor maintenance but also for the initiation of distant metastases.

More recently, GC-PDCOs have emerged as indispensable tools for parsing the complex genetic drivers of morphological transformation, particularly in the context of diffuse-type gastric cancer (DGC) (Tong et al., 2023). By utilizing CRISPR-Cas9-mediated gene editing or selecting for specific clinical mutations, researchers have elucidated how the loss of CDH1 and the gain of oncogenic RHOA mutations synergize to drive epithelial-to-mesenchymal transition (EMT) and amoeboid-like invasive behavior (Schaefer et al., 2023). These models have revealed that RHOA mutations are critical for achieving anchorage-independent growth and escaping anoikis, two hallmarks of metastatic progression in DGC (Zhou et al., 2014). Beyond modeling initial drivers, CRISPR-Cas9-mediated gene editing provides a powerful framework for simulating therapy-induced “clonal evolution.” By introducing specific mutations or knockouts into established organoid lines, researchers can track how sub-clones harboring CDH1 loss or RHOA variants selectively expand under pharmacological pressure (Li et al., 2025a). This capability transforms PDCOs into a dynamic platform for capturing lineage drift and the emergence of resistant sub-populations, offering high-resolution insights into the adaptive mechanisms and acquired resistance that characterize the evolutionary trajectory of gastric cancer during treatment (Sáenz et al., 2022; Sheng et al., 2026). The technological refinement of these models has also extended to identifying novel functional markers like AQP5 (Aquaporin-5), which robustly enriches for cancer stem cells (CSCs) across mouse and human tumors, and LRP8, which serves as a molecular bridge facilitating *H. pylori* (*Helicobacter pylori*)-induced β-catenin nuclear translocation (Lim et al., 2025). Collectively, these milestones have bridged the gap between static genomic sequencing and dynamic functional analysis, positioning GC-PDCOs as high-fidelity models for exploring the mechanistic underpinnings of gastric carcinogenesis (Albrecht et al., 2025).

### 2.4 Standardized workflow and quality control for GC-PDCO preparation

Given the technical difficulties and variable success rates in establishing GC-PDCOs, a standardized operational workflow is essential for achieving reproducibility and establishing reliable living biobanks (Yang et al., 2024a). The typical workflow begins with tissue procurement and transport. Studies indicate that surgical resections or endoscopic biopsies can be shipped in standard cold storage solutions (such as HBSS or UW solution) at 4 °C for up to 48 h without significantly compromising subsequent organoid viability, growth rates, or downstream drug response profiles (Skubleny et al., 2023).

For tissue processing, samples are digested into single-cell suspensions or isolated gastric glands using chemical chelation or mixed enzymatic digestion (Jones et al., 2021; Buckley et al., 2024). These isolated components are subsequently seeded into three-dimensional extracellular matrices, most commonly Matrigel, and cultured in chemically defined media tailored to maintain gastric stem cell niches (Jones et al., 2021). Implementing a standardized initial cell seeding density has been shown to consistently yield robust organoids, although formation efficiency may inherently vary based on the anatomical origin; for instance, antral biopsies often display more rapid initial growth compared to corpus biopsies (Buckley et al., 2024).

During the expansion phase, GC-PDCOs can be serially passaged through either mechanical fragmentation or single-cell enzymatic dissociation (Jones et al., 2021; Buckley et al., 2024). These organoids exhibit robust long-term proliferation and can be successfully cryopreserved and thawed while maintaining their structural fidelity, cellular polarity, and patient-specific genomic profiles (Jones et al., 2021; Miao et al., 2022).

To mitigate inter-laboratory variability and ensure translational reliability, the implementation of a rigorous Quality Control (QC) framework is highly recommended. Recent consensus guidelines propose standardizing PDO characterization by verifying a “tripartite” consistency: histopathological concordance, genomic stability, and functional drug response correlation with the parental tumor (Xiang et al., 2024; Yang et al., 2024a). Furthermore, novel non-destructive evaluation technologies are emerging to support standard QC. For example, AI-driven image analysis algorithms (e.g., CBAM-YOLOv3) have been successfully developed to precisely monitor organoid vitality and cellular senescence based on parameters like organoid diameter and SA-β-gal staining intensity, providing an automated and quantitative metric for managing clinical-grade organoid biobanks (Yang et al., 2023).

## 3 Modeling the gastric TME and immune evasion

### 3.1 Simulation of the three major mechanisms of immune escape

The convergence of multi-dimensional patient-derived models has enabled a high-resolution dissection of the mechanisms facilitating immune evasion in gastric cancer, which can be strategically categorized into a tripartite framework: Camouflage, Coercion, and Cytoprotection (Kan et al., 2025; Galassi et al., 2024). Camouflage serves as the primary layer of defense. High-fidelity GC-PDCO models reveal that tumor cells often exploit intrinsic defects in their antigen processing and presentation machinery, such as the downregulation or shedding of MHC-I molecules, to effectively render themselves invisible to immunosurveillance (Kan et al., 2025; Prakash et al., 2026; Hosseini-Farahabadi et al., 2026). Beyond cellular-level genetic loss, the methodological advancement of ALI organoid systems has successfully preserved native extracellular matrix (ECM) components. These models demonstrate a common macroscopic pattern: the ECM forms a dense physical stromal barrier that prevents the productive infiltration of cytotoxic CD8^+^ T lymphocytes into the malignant core, thereby sequestering tumor-associated antigens (He et al., 2025; Yao et al., 2023).

When camouflage fails and immune recognition occurs, tumor cells transition to Coercion, a proactive mechanism designed to suppress or exhaust infiltrating immune effectors. The application of human tumor-infiltrating lymphocyte and organoid (huTGO) co-culture systems has robustly mapped the aberrant expression of inhibitory ligands, such as programmed death-ligand 1 (PD-L1), with high clinical concordance (Chakrabarti et al., 2021). In HER2-positive gastric cancer, these co-culture models have elucidated a critical immunosuppressive pattern where HER2 amplification directly drives PD-L1 upregulation, effectively “coercing” PD-1-expressing T cells into a state of functional exhaustion (Suh et al., 2017). This immunosuppressive state is further stabilized by the recruitment of myeloid-derived suppressor cells and novel regulatory factors (e.g., TRIM28, specific circular RNAs), which collectively blunt the efficacy of immune checkpoint inhibitors (Kan et al., 2025; Tyrinova et al., 2024; Mabrouk et al., 2023; Ma et al., 2023).

**FIGURE 2:**
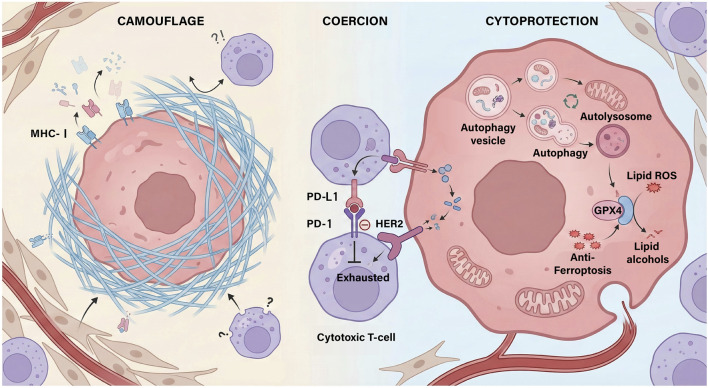
The “3C model” of immune evasion mechanisms in gastric cancer.

Finally, tumors employ Cytoprotection to bolster their resilience against immune-mediated cell death pathways. Single-cell RNA sequencing of therapy-resistant organoid lineages has identified the adaptive upregulation of autophagy—characterized by the increased formation of autophagy vesicles and autolysosomes—as a common factor in evading therapy-induced stress (Yang et al., 2025). The functional necessity of this mechanism is particularly evident in models derived from gastric cancer peritoneal metastasis (GCPM) ascites, where high-plasticity GC clusters undergo an autophagy-dependent plasticity shift to evade therapy, making them vulnerable to targeted autophagy inhibitors (Huang et al., 2023). In parallel, PDCO platforms reveal that GC cells exploit metabolic reprogramming to evade non-apoptotic cell death, specifically ferroptosis (Galassi et al., 2024). Research has demonstrated that resistant PDCO lineages upregulate anti-ferroptotic metabolic “shields” (such as glutathione-dependent pathways and GPX4) to buffer against the accumulation of lipid reactive oxygen species (ROS), thereby maintaining redox homeostasis and resisting immune-induced death (Wang et al., 2024; Rosell et al., 2023) (Figure 2).

### 3.2 Interaction with interstitium and immune components

Beyond tumor-intrinsic mechanisms, the structural and biochemical interplay between GC cells and their surrounding stroma provides a critical secondary layer of cytoprotection (Wang et al., 2025b; Calice et al., 2025). A significant limitation of traditional epithelial-only PDCOs is the exclusion of the complex stromal and immune niches. To overcome this disadvantage and systematically study the TME, researchers currently employ three primary bioengineered co-culture methodologies (Song et al., 2024; Chen et al., 2026b).

First, reconstituted co-culture systems (including assembloids) involve the controlled combination of purified tumor organoids with specific exogenous or autologous cell populations. By integrating tumor epithelial cells with cancer-associated fibroblasts (CAFs), mesenchymal stem cells (MSCs), endothelial cells, or immune effectors (such as macrophages and natural killer cells) in direct contact or via Transwell systems, researchers can precisely dissect specific cell-to-cell crosstalk, such as CAF-induced NK cell ferroptosis or macrophage M2 polarization (Yao et al., 2023; Shapira-Netanelov et al., 2025; Yang et al., 2024b; Ding et al., 2022). Second, Air-Liquid Interface (ALI) holistic models preserve the native TME architecture by culturing mechanically minced tumor fragments without full enzymatic single-cell dissociation. This approach physically retains endogenous tumor-infiltrating lymphocytes (TILs), CAFs, and native extracellular matrix components, providing an intact immune landscape highly suitable for evaluating immune checkpoint inhibitors and tracking immune evasion (Zheng et al., 2025a; Takeuchi et al., 2023; Xing et al., 2026). Third, microphysiological systems integrate organoids into microfluidic devices to introduce dynamic physical cues—such as continuous perfusion and fluid shear stress—enabling the spatial compartmentalization of tumor cells and immune/stromal niches to simulate systemic vascular delivery and complex immune cell trafficking (Huang et al., 2024; Heninger et al., 2021).

It is precisely through the application of these highly integrated models that researchers have been able to capture phenomena entirely unobservable in monoclonal PDOs, such as the marked upregulation of pro-inflammatory cytokines and stroma-mediated drug resistance. Comparative transcriptomic analyses have explicitly demonstrated this discrepancy: compared to epithelial monocultures, patient-specific assembloids show markedly higher expression of inflammatory cytokines and ECM remodeling factors across different stromal ratios (Shapira-Netanelov et al., 2025).

A critical pattern identified through these integrated systems is the profound shift in therapeutic sensitivity: while certain chemotherapies and targeted agents remain highly effective in monocultured epithelial organoids, they frequently lose efficacy within matched assembloid models (Shapira-Netanelov et al., 2025; Lyu et al., 2025; Nam et al., 2025). CAFs, the most prominent stromal constituent, act as primary architects of this acquired resistance. PDO-CAF co-culture units have demonstrated that CAFs form extensive networks surrounding tumor organoids, conferring robust resistance to standardized regimens (e.g., 5-FU, paclitaxel) through two main avenues (Yin et al., 2025; Uematsu et al., 2025). This dynamic is profoundly influenced by the intrinsic histological subtype; for example, monocultured poorly cohesive carcinoma (PCC) organoids may demonstrate heightened sensitivity to docetaxel in isolation, but CAF co-culture overwhelmingly rescues these cells and confers broad-spectrum chemoresistance to multiple agents (Yin et al., 2025). Mechanically, myofibroblastic CAFs modulate matrix stiffness, increasing mechanical tension that triggers survival signaling pathways in GC cells (Lu et al., 2023; Ozmen et al., 2024; Jang et al., 2018). Biochemically, the stroma secretes soluble inhibitory factors and non-coding RNAs (e.g., circ_0008315) that directly enhance cancer stem cell properties (Uematsu et al., 2025; Lu et al., 2023; Ozmen et al., 2024; Fei et al., 2024).

Furthermore, assembloid models reveal that the GC tumor microenvironment is not merely a static physical barrier but a dynamic participant in driving immune exclusion (Wei et al., 2023; Zhao et al., 2023b). Assembloids containing CAFs and MSCs show marked upregulation of immunosuppressive signaling (e.g., IL-10, IL-17) and create a “metabolic shielding barrier” (Shapira-Netanelov et al., 2025; Xu et al., 2024b; Wang et al., 2023). By intensely competing for essential nutrients and releasing suppressive metabolic byproducts, the stroma drives local immune cells into functional exhaustion (Yin et al., 2025; Zhang et al., 2026a). Ultimately, assessing therapeutic responses necessitates acknowledging spatial and lineage heterogeneity. Single-cell comparisons between primary tumors and matched PDOs have demarcated the molecular boundaries of these experimental models, confirming that while PDO biobanks successfully capture regional heterogeneity and subclonal architecture, their predictive accuracy relies heavily on integrating the diverse stromal and spatial components present in the original lesion (Kumar et al., 2022; Yan et al., 2018). By recapitulating these intricate tumor-stroma-immune interactions, patient-derived assembloids offer a much more physiologically relevant platform for evaluating clinical drug responses than traditional monocultures.

### 3.3 Coupling of intragastric microecology and tumorigenesis

Complementing the structural complexity of the TME is the intricate intragastric microecology. The stomach is a dynamic niche where the coupling of microbial dysbiosis and host signaling dictates the “inflammation-cancer” transition (Liu et al., 2025b). Gastric cancer organoids (GCOs) have revolutionized this field, with the maturation of luminal microinjection technology serving as a definitive milestone. By precisely injecting pathogens directly into the enclosed organoid lumen, researchers can now authentically mimic the natural polarity of apical infection and the subsequent breakdown of the epithelial barrier (Liu et al., 2025c). This specific technical breakthrough has been instrumental in shifting the paradigm, explicitly unveiling the non-mutational mechanisms by which microbes drive tumorigenesis.

*H. pylori* remains the primary orchestrator of this dysbiotic environment. Instead of merely inducing chronic inflammation, organoid models have elucidated common non-mutational mechanisms by which it drives oncogenesis, such as hyperactivating stem cell pathways (e.g., CDK1/β-catenin) and directly inducing genomic instability via DNA repair suppression, driving widespread field cancerization (Zhu et al., 2023; Xu et al., 2025).

Beyond *H. pylori*, GCO co-culture models have demonstrated that the microenvironment becomes permissive to the translocation of oral and intestinal microbiota (e.g., *Fusobacterium* nucleatum), which independently enhance barrier disruption and innate immune signaling (Liu et al., 2025b; Dong et al., 2017). (Lin et al., 2025). The utility of the organoid platform is particularly evident in modeling multi-pathway interactions, such as co-infections (e.g., *H. pylori* and Epstein-Barr virus) or the synergistic effects of environmental heavy metals. These models consistently show that microbial shifts and toxins act synergistically to accelerate tissue morphogenesis, hyperproliferation, and oxidative stress (Liu et al., 2025c; Reytor-González et al., 2025). Crucially, insights from high-fidelity GCOs confirm that the intragastric microecology directly fuels the “3C model” of immune evasion (Kan et al., 2025; Chakrabarti et al., 2021). Pathogen-induced barrier breakdown facilitates early-stage “Camouflage”, while microbial metabolites (such as lactic acid from dysbiotic flora) contribute to “Coercion” and “Cytoprotection” by acidifying the niche, suppressing immune effectors, and promoting a hypochlorhydric environment (Liu et al., 2025b; Zhang et al., 2026b). This underscores the necessity of managing the gastric TME not just as a collection of human cells, but as a complex ecosystem where microbial coupling actively orchestrates the immunosuppressive landscape.

## 4 PDCO-guided functional precision oncology

### 4.1 Prediction of sensitivity to chemotherapy and radiotherapy

The clinical implementation of GC-PDCOs represents a transformative milestone in functional precision oncology, transitioning the technology from a descriptive modeling tool to a predictive “patient avatar” for therapeutic decision-making (Skubleny et al., 2025; Song et al., 2016). Traditional therapeutic selection for GC has long been hampered by a lack of reliable biomarkers, with standard neoadjuvant and palliative regimens often yielding complete or partial responses in only a minority of patients (Derieux et al., 2020). GC-PDCOs address this bottleneck by providing a rapid, high-throughput platform for *ex vivo* drug sensitivity testing (DST) that faithfully mirrors the pharmacological landscape of the parent tumor (Gao et al., 2018). Early prospective co-clinical studies have demonstrated that these models exhibit a strong degree of clinical concordance. For instance, testing with conventional cytotoxic agents like the FLOT regimen components has yielded high sensitivity and specificity in predicting patient responses within limited exploratory cohorts (Schmäche et al., 2024). However, it is important to note that these promising results are currently derived from studies with relatively small sample sizes, underscoring the need for large-scale, multicenter validation before universal clinical adoption can be achieved. Notably, landmark research utilizing PDCOs from metastatic gastrointestinal cancer patients reported a 100% sensitivity and 93% specificity in predicting clinical response or resistance, effectively identifying non-responders who would not benefit from standard-of-care regimens. The selection of Drug Sensitivity Testing (DST) evaluation indicators is strategically aligned with the pharmacological mechanisms of the administered agents to ensure predictive consistency. While metabolic viability assays provide a primary readout, standardized thresholds for structural disintegration and specific apoptotic signatures are increasingly utilized to recapitulate *in vivo* pathological regression (Li et al., 2025b). By integrating these functional indicators with molecular stratification, clinicians can transition from binary sensitivity results to a multidimensional assessment that correlates drug-induced phenotypic changes with the patient’s unique genomic landscape.

This high functional fidelity is particularly critical in the neoadjuvant setting, where the timeframe for intervention is narrow. GC-PDCOs can be successfully established from endoscopic biopsies within 7–14 days, a period that aligns seamlessly with the routine clinical staging workflow involving CT scans and surgical planning (Skubleny et al., 2025). This rapid turnaround allows for real-time decision support for neoadjuvant chemotherapy or chemoradiotherapy, enabling clinicians to pivot toward more effective regimens like FLOT or targeted combinations before the initiation of treatment (Song et al., 2016). Morphological and histological analyses have confirmed that GC-PDCOs accurately recapitulate the cell death patterns observed *in vivo*; for instance, exposure to paclitaxel and oxaliplatin in sensitive organoid lines results in distinct structural disintegration and apoptotic signatures that parallel the pathological regression seen in surgical specimens (Wallaschek et al., 2019). Furthermore, the integration of molecular stratification, such as using the 107-gene Nanostring assay to map TCGA and TME subtypes, enhances the predictive power of these models by linking functional drug responses to specific genomic landscapes.

Beyond direct drug screening, GC-PDCOs facilitate the identification of novel biomarkers for resistance and recurrence. Recent studies have highlighted the role of peptidase inhibitor 3 (PI3) expression in predicting 5-FU resistance and subsequent disease-free survival, while identifying the P23/HSP90/XRCC1 signaling axis as a critical node for modulating DNA damage repair and sensitivity to novel natural compounds like ailanthone (Harada et al., 2025). These insights allow for the development of “synthetic lethality” strategies where multiple signaling pathways are targeted simultaneously based on the organoid’s functional profile. While challenges regarding intratumoral heterogeneity and the exclusion of complex stromal interactions remain, the use of PDCO-guided screening provides a dynamic, functional readout that far surpasses the predictive capacity of static genomic sequencing, ultimately moving the field closer to truly individualized management of every gastric cancer patient (Albrecht et al., 2025) (Table 1).

**TABLE 1:** Summary of patient-derived organoid (PDO) studies in functional precision medicine for gastric cancer.

The number of organoids	Therapeutic regimens	Key methodology	Main discovery	Key references
Esophago-gastric cancer (N = 45)	5-FU, Oxaliplatin, Docetaxel (FLOT)	PDO-based drug response thresholds	Successfully predicted pathological response to neoadjuvant chemotherapy with high clinical correlation.	Schmäche et al. (2024)
Gastric cancer (N = 33)	Various targeted agents based on TCGA subtypes	107-gene Nanostring Assay + PDO screening	Effectively translated TCGA and TME classifications into PDO models to guide subtype-specific drug selection.	Skubleny et al. (2025)
Various GI cancers (N = 9)	5-FU, Oxaliplatin, Irinotecan, and targeted therapies	Integration of genomics and circulating tumor cell organoids	Demonstrated that CTOs can be established from blood to predict real-time clinical response when surgical tissue is unavailable.	Wu et al. (2024)
Gastric cancer (N = 50)	DNA-damaging drugs (5-FU, Oxaliplatin)	RNA-seq of PDOs + clinical recurrence analysis	Identified PI3 expression as a key predictor of recurrence; PI3-high PDOs showed resistance to DNA-damaging agents, matching clinical outcomes.	Harada et al. (2025)
Gastric cancer (multicenter)	Cisplatin and immunotherapy related drugs	tsRNA-defined molecular subtyping + PDO validation	Identified three tsRNA-based subtypes that correlate with specific drug sensitivities, providing a roadmap for precision oncology.	Tian et al. (2025)

### 4.2 Strategy optimization for targeted therapy

The clinical management of advanced GC has transitioned from a one-size-fits-all cytotoxic approach to a biomarker-driven paradigm, yet the high rate of primary and acquired resistance necessitates the use of PDCOs to optimize targeted strategies. HER2 remains the most established therapeutic node, where Trastuzumab is the standard first-line treatment for HER2-positive cases (Albrecht et al., 2025; Bang et al., 2010). PDCOs have proven instrumental in modeling the divergent responses observed in the clinic; for instance, while Trastuzumab significantly reduces viability in HER2-amplified organoid lines, its efficacy is often hampered by downstream signaling bypasses, such as the activation of the MAPK pathway (Tian et al., 2025). Research utilizing spatial transcriptomics and PDCOs has identified two major independent mechanisms of Trastuzumab resistance: the induction of the EMT in approximately one-third of patients and the activation of the endoplasmic reticulum-associated degradation pathway in another third (Sheng et al., 2026). Specifically, HER2 blockade can trigger a transcriptional shift toward a mesenchymal state characterized by the upregulation of ZEB1/2 and immune exhaustion markers like PD-L1, suggesting that combining HER2 inhibitors with immune checkpoint blockade may rescue sensitivity in these “immune-cold” mesenchymal-high subsets (Sheng et al., 2026). Furthermore, the Wnt/β-catenin signaling pathway has been identified as a critical driver of acquired Trastuzumab resistance, as resistant PDCOs exhibit a stemness-like phenotype that can be partially reversed by Wnt3a depletion (Kim et al., 2023).

To overcome Trastuzumab-refractory disease, the antibody-drug conjugates (ADCs) Trastuzumab deruxtecan (T-DXd) has emerged as a potent second-line option (Shitara et al., 2024). PDCO-based sensitivity assessments reveal that T-DXd maintains high tumoricidal activity even in samples with heterogeneous HER2 expression, though resistance eventually develops through HLA transcriptional loss and the upregulation of oxidative phosphorylation pathways (Sheng et al., 2026). Innovative strategies to enhance HER2 targeting now include the use of bispecific antibodies, such as IBI315, which targets both PD-1 and HER2 (Lin et al., 2023). PDCO co-culture models with autologous T cells have demonstrated that IBI315 facilitates the formation of immune synapses and triggers Gasdermin B (GSDMB)-mediated pyroptosis in tumor cells, creating a positive feedback loop via interferon-gamma (IFN-γ) secretion that further upregulates GSDMB and PD-L1, effectively turning “cold” tumors “hot” (Lin et al., 2023).

Emerging targets such as Claudin 18.2 (CLDN18.2) and FGFR2b are also being integrated into the PDCO-guided precision pipeline. Zolbetuximab, an anti-CLDN18.2 antibody, has shown significant efficacy in first-line settings for HER2-negative disease (Shitara et al., 2023; Shah et al., 2023). Interestingly, spatial profiling of HER2-positive tumors following Trastuzumab resistance has revealed a significant upregulation of CLDN18.2 expression, increasing from 16.7% in treatment-naïve samples to 50% post-progression (Sheng et al., 2026). This dynamic expression profile suggests a clear rationale for sequential or combined targeting of HER2 and CLDN18.2 to circumvent escape mechanisms. Consequently, longitudinal monitoring using organoid platforms can serve as a functional sentinel to identify the optimal switch point between HER2-targeted and CLDN18.2-targeted regimens, optimizing the timing of sequential therapies to circumvent acquired resistance (Sheng et al., 2026). Similarly, for the subset of patients (10%–30%) exhibiting FGFR2b overexpression or amplification, the monoclonal antibody Bemarituzumab has demonstrated a median overall survival benefit in Phase 2 trials (Smyth et al., 2022). PDCO biobanks covering these molecular subtypes allow for high-throughput screening of FGFR tyrosine kinase inhibitors (TKIs) like Futibatinib and Erdafitinib, identifying specific vulnerabilities in FGFR2-amplified lineages.

**FIGURE 3:**
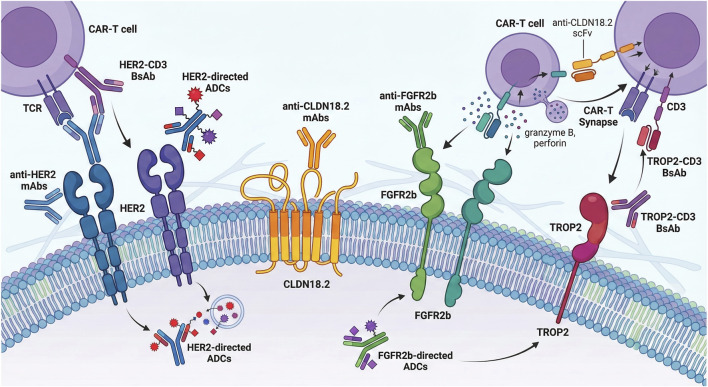
The therapeutic landscape of precision oncology in gastric cancer.

Beyond these primary receptors, PDCOs are facilitating the exploration of broader signaling nodes, including the KRAS-MAPK axis and niche-independent growth mechanisms. Activation of oncogenic KRAS^G12D^ has been shown in organoid models to drive niche escape by inducing autonomous epithelial WNT secretion, specifically WNT7B, via a MAPK-TGFBRII-SMAD2/3 signaling axis (Lee et al., 2025). This finding highlights a potential therapeutic vulnerability, where Porcupine inhibitors or MAPK inhibitors can be used to arrest the growth of WNT-independent gastric tumors (Lee et al., 2025). Additionally, synergistic combinations targeting the JAK2/STAT3 pathway, such as the STAT3 inhibitor Napabucasin and the p53 agonist COTI-2, have shown enhanced tumor-killing effects in PDCO models of advanced progressive gastric cancer (Xu et al., 2024a). By integrating these functional readouts with multi-omics characterization, PDCO-guided platforms are evolving from simple sensitivity assays into sophisticated tools for decoding the complexity of therapeutic evasion in gastric cancer (Figure 3).

### 4.3 Personalized assessment of immunotherapy

The paradigm shift toward immunotherapy in gastric cancer has necessitated the development of functional platforms capable of predicting individual responses, as traditional biomarkers such as PD-L1 combined positivity score and MSI status often fail to capture the dynamic nature of tumor-immune interactions. PDCOs have emerged as essential “immuno-avatars” when integrated into advanced co-culture systems with autologous immune cells, allowing for the *ex vivo* evaluation of ICIs, adoptive cell therapies, and novel biological agents (Chiriaco et al., 2022). Recent research utilizing the POLARSTAR trial framework has demonstrated that GC-PDCOs can effectively model the synergistic relationship between radiotherapy and PD-1 blockade (Pang et al., 2025). On a cellular level, ionizing radiation induces GSDMD-mediated pyroptosis in colorectal and gastric cancer cells, a form of immunogenic cell death that enhances the infiltration of cytotoxic T cells into the tumor microenvironment (Pang et al., 2025). By testing these combinations in PDCO-immune co-cultures, clinicians can functionally validate the “*in situ* vaccine” effect of localized treatments, identifying patients most likely to benefit from the artificial conversion of “cold” tumors into “hot,” immune-responsive ones.

Beyond checkpoint inhibition, PDCO platforms provide a high-resolution testbed for the killing efficiency of chimeric antigen receptor (CAR)-based therapies, such as CAR-T and CAR-NK cells (Chiriaco et al., 2022). For instance, MET-CAR-T cells have demonstrated specific, dose-dependent cytotoxic activity against MET-overexpressing gastric cancer models, including 3D organoids. Functional assays have shown that MET-CAR-T cells disrupt patient-derived organoids more efficiently than non-transduced T cells, with cell killing directly proportional to the surface density of the target antigen. This allows for the establishment of a “killing threshold,” sparing normal tissues with physiological MET expression while robustly targeting malignant cells, even those harboring resistance to traditional TKIs (Chiriaco et al., 2022). Similarly, PDCOs are being utilized to evaluate the tumoricidal potential of oncolytic viruses and bispecific antibodies, providing a dynamic readout of immune synapse formation and subsequent tumor lysis. These models enable the measurement of granzyme B and IFN-γ secretion following antigen engagement, offering a comprehensive assessment of T-cell activation and the resulting inflammatory response within a patient-specific context.

Finally, the integration of multi-omic stratification with functional PDCO testing is refining patient stratification for personalized immunotherapy (Tian et al., 2025). Novel molecular subtyping based on transfer RNA-derived small RNAs (tsRNAs) has identified distinct immune-related clusters, such as the Stromal_H and Stromal_L subtypes, which exhibit divergent clinical outcomes (Tian et al., 2025). Patients stratified into the low-risk “GCtsRNAscore” group exhibit a 2.3-fold higher objective response rate to immunotherapy compared to high-risk groups, who may instead benefit more from targeted therapies like axitinib or dasatinib. By performing functional drug and immune cell screenings on PDCOs derived from these molecularly defined cohorts, clinicians can prospectively match patients to the most effective therapeutic modality (Xu et al., 2024a; Schmäche et al., 2024). This functional precision oncology approach moves beyond static genomic sequencing, providing real-time evidence of therapeutic efficacy and bridging the gap between complex tumor microenvironment signatures and individualized clinical management (Albrecht et al., 2025).

### 4.4 Photodynamic therapy (PDT) and microbial-assisted therapy

Complementing the advancements in immunotherapeutic evaluation, GC-PDCOs are increasingly utilized to refine light-activated and microbially-modulated therapeutic strategies, offering a unique platform to evaluate modalities that rely on the spatial organization of the tumor mass (Jeong et al., 2025). PDT has emerged as a promising, minimally invasive modality for treating early-stage gastric cancer, and PDCOs provide a superior 3D architecture for testing the delivery and tumor-killing efficiency of photosensitizers (Cao et al., 2023). Recent research has successfully employed PDCO platforms to investigate the efficacy of novel indocyanine green-loaded nanomaterials under near-infrared irradiation. These models have proven essential for quantifying photosensitizer uptake and subsequent ROS generation within a spatially organized malignant structure, a feat often unachievable in traditional 2D systems (Panikar et al., 2019). Furthermore, the integration of advanced imaging, such as light-sheet fluorescence microscopy with gastric organoids, allows for the real-time visualization of cellular dynamics and secretory functions in response to photo-activated cues, thereby facilitating the optimization of light penetration and treatment dosimetry for individual patients (Yao and Smolka, 2019). Crucially, the PDCO platform serves as a high-precision interface for establishing clinical intervention thresholds. By utilizing matched normal gastric units as a baseline, researchers can precisely titrate photosensitizer dosages or microbial metabolite concentrations based on individualized pharmacological response curves (Schmäche et al., 2024). This *ex-vivo* titration, supported by real-time monitoring of reactive oxygen species production, allows for the quantification of a therapeutic window that maximizes tumoricidal effects while strictly remaining below the toxicity threshold for normal mucosa, ensuring the safety and efficacy of these spatially-organized therapies (Weng et al., 2023).

Simultaneously, the emerging paradigm of “precision oncomicrobiology” leverages PDCOs to dissect the complex role of the intragastric microbiota in modulating anti-tumor responses and malignant transformation. While traditional probiotics such as *Lactobacillus* have been noted for their potential anti-inflammatory properties against *H. pylori* infection (Mentis et al., 2019), high-fidelity organoid-based co-culture models and INS-GAS mouse studies have revealed a more nuanced, “double-edged” interaction within the gastric microenvironment. For instance, deoxycholic acid-induced enrichment of the *Lactobacillus* genus, particularly *Lactobacillus* johnsonii, has been shown to actually accelerate gastric atrophy and intestinal metaplasia (IM) (Jin et al., 2022). Mechanistically, these microbes can metabolize lactose into lactic acid, which acidifies the gastric mucous layer and subsequently inhibits gastrin secretion by antral G cells, thereby promoting a hypochlorhydric environment permissive to further neoplastic progression (Myllyluoma et al., 2007). PDCO platforms enable the functional screening of microbial metabolites, such as short-chain fatty acids, and their specific effects on different cell types in the gastric gland (Idowu et al., 2022). By integrating photo-activated sensitivity assays with such microbial co-cultures, PDCOs facilitate the design of personalized “assisted” therapeutic regimens that can fine-tune the microbial milieu to suppress oncogenic transitions while enhancing the efficacy of standard clinical interventions.

## 5 Clinical translation: opportunities and workflow

### 5.1 Current status of clinical trials

The transition of GC-PDCOs from the laboratory to clinical practice is currently defined by an increasing volume of registered clinical trials and co-clinical studies designed to validate their prospective utility. Currently, the most robust evidence for the role of PDCOs in improving clinical outcomes, such as progression-free survival (PFS) and overall survival (OS), stems from their use as “reverse translation” models within the framework of large-scale Phase III trials. A landmark study in this field involved the integration of PDOs into the REAL3 trial population, which evaluated the addition of panitumumab to the EOX (epirubicin, oxaliplatin, and capecitabine) regimen (Smyth et al., 2021). By modeling the interaction between the chemotherapy backbone and EGFR inhibitors in organoids, researchers identified a paradoxical antagonistic effect between epirubicin and anti-EGFR agents specifically in EGFR-amplified cases (Smyth et al., 2021). This antagonistic interaction provided a biological rationale for the clinical results, where patients with EGFR-amplified tumors treated with panitumumab exhibited a lower median OS of 9.74 months compared to 11.18 months in non-amplified cases. Such findings underscore the capacity of PDCOs to prevent the implementation of detrimental therapeutic combinations, thereby directly safeguarding patient survival through refined trial design (Mirlohi et al., 2025; Martin et al., 2025).

Beyond identifying drug antagonisms, PDCOs are instrumental in the preclinical validation of novel agents currently entering Phase I and II trials. For instance, the development of SYSA1801, a potent ADC targeting CLDN18.2, relied heavily on GC-PDO models to demonstrate superior tumoricidal activity, providing the foundational evidence for its ongoing clinical evaluation (Egebjerg et al., 2026; Luo et al., 2026). Similarly, organoid-based platforms have been registered to identify drivers of resistance to standard-of-care agents; the identification of IGFBP2 as a mediator of platinum resistance and the role of TDO2 in maintaining CSCs viability have led to the design of trials that utilize these markers to stratify patients for adjunct therapies (Pham et al., 2023). The clinical relevance of these models is further highlighted in refractory subtypes like the SEM (stem-like/EMT/mesenchymal) type, where PDCOs identified a hidden metabolic vulnerability in Caveolin-1 (CAV1)-mediated endocytosis (Hwang et al., 2023). Clinical cohort analyses have correlated high CAV1 expression with significantly poorer overall survival, but subsequent PDO validation demonstrated that lysosomal inhibitors such as chloroquine can successfully block this survival mechanism, offering a potential adjunct to standard chemotherapy to extend PFS in treatment-refractory patients (Hwang et al., 2023).

The next Frontier of clinical translation involves interventional trials where PDCO-derived DST results actively guide the selection of third-line and salvage therapies. Preliminary data from these frameworks suggest that organoid-guided treatment shows strong potential for clinical concordance, particularly in predicting resistance to conventional regimens like 5-FU and oxaliplatin, though these findings await confirmation in larger patient populations. This is especially critical for high-risk clinical scenarios such as gastric cancer peritoneal metastasis, where the loss of function in Trp53 and Cdh1 often leads to rapid paclitaxel resistance and a dismal prognosis with survival often limited to 6 months (Liu et al., 2025d). PDCO models have identified Src inhibitors like dasatinib as effective agents for delaying disease progression in these specific genetic backgrounds, providing a strategy to ameliorate clinical manifestations and prolong OS in a population with limited therapeutic options (Liu et al., 2025d). As standardized pipelines for high-throughput screening and autologous immune co-cultures continue to mature, the prospective integration of PDCOs into routine clinical workflows promises to reduce the administration of ineffective toxicities and ultimately realize the survival benefits of functional precision oncology (Baisiwala et al., 2026; Yu, 2025) (Table 2). Despite these advancements, a critical “translational gap” remains, as high-fidelity DST may still fail to predict overall survival due to systemic factors and sampling errors. For instance, in mucinous or signet-ring cell subtypes, superficial biopsies may lack sufficient representation of the infiltrative tumor core, leading to potential predictive bias (Zheng et al., 2025b). Furthermore, spatial heterogeneity across multi-focal lesions and systemic metabolic variations can cause *ex-vivo* pharmacological responses to deviate from actual clinical outcomes (Wu et al., 2025). Acknowledging these limitations is essential for clinicians to interpret PDCO results objectively and underscores the necessity for multi-site sampling and standardized protocols to bridge the gap between bench-side modeling and bedside reality.

**TABLE 2:** The current status of clinical trials related to gastric cancer organoids.

NCT number	Phase	Research model	Key intervention	Findings
NCT00824785	Phase III	Advanced gastro-oesophageal adenocarcinoma tissue and patient-derived organoids	EOX (epirubicin, oxaliplatin, capecitabine) ± panitumumab	In EGFR-amplified PDOs, EGFR inhibitors paradoxically increased viability by antagonizing epirubicin through p21/cyclin B1 downregulation and cyclin E1 upregulation, which accelerated the cell cycle.
NCT02695459	Phase II	Gastroenteropancreatic neuroendocrine carcinoma (GEP-NEC) PDOs	Cisplatin and everolimus	The overall establishment rate for GEP-NEC organoids was 16%, and the drug sensitivity of the PDOs was found to parallel the actual clinical responses (PR/SD/PD) of the patients.
ChiCTR2100047129	Phase I	Advanced gastric cancer (GC) patients and PDOs	IMC001 (autologous EpCAM-targeted CAR-T cell therapy)	IMC001 achieved a 40% confirmed objective response rate (ORR) at the middle-dose level; PDO models confirmed a safety window as killing efficiency was significantly higher in cancerous vs. normal tissue organoids.
NA	Phase II	Type 1 gastric neuroendocrine tumors tissue and mouse gastric organoids	Netazepide (YF476, a gastrin/CCK2R antagonist)	Netazepide inhibited gastrin-induced PAPPA2 expression, which decreased IGF bioavailability, thereby suppressing cell migration and tissue remodeling involved in gNET development.

### 5.2 Functional precision oncology in clinical practice

The transition from static genomic profiling to functional precision oncology (FPO) hinges on the ability of GC-PDCOs to serve as dynamic, prospective decision-support tools. Unlike the retrospective nature of genomic sequencing, which identifies potential therapeutic vulnerabilities through a “lookup table” of mutations, FPO utilizes PDCOs to directly measure pharmacological outcomes across a wide spectrum of standard-of-care and investigational agents (Mircetic et al., 2023). The successful clinical implementation of this paradigm requires a rigorously standardized workflow that ensures reproducibility while meeting the narrow temporal window demanded by advanced gastric cancer management. Recent studies have demonstrated that GC-PDCO biobanks can predict clinical responses to 5-FU, oxaliplatin, and cisplatin with high accuracy, yet the “scalability-fidelity” trade-off remains a significant barrier to universal adoption (Zhao et al., 2024; Schmäche et al., 2024). Establishing standardized protocols for human gastric organoid culture, such as those emphasizing chemically defined media and mechanical versus enzymatic dissociation, is the foundational step in minimizing the inter-laboratory variability that has historically plagued preclinical models (Mircetic et al., 2023).

A critical bottleneck in the FPO pipeline is the achievement of “clinically actionable timeframes,” where therapeutic guidance must be delivered within 10–14 days of biopsy to prevent patient deterioration or the initiation of suboptimal empirical treatment (Mircetic et al., 2023). To navigate the challenge of balancing model complexity with this narrow window, the workflow must prioritize the integration of “augmented organoids” with high-speed analytical technologies. The implementation of real-time imaging and machine-learning-assisted viability assays can detect therapeutic responses several days earlier than conventional metabolic assays, effectively shortening the expansion cycle (Jones et al., 2026). Furthermore, transitioning to micro-organoid formats and high-throughput automated platforms allows for the maintenance of high-fidelity tumor-stroma-immune interactions while strictly adhering to the 10–14-day clinical decision-making deadline (Du et al., 2025). Current efforts to fast-track this workflow involve the optimization of initial expansion phases, moving away from low-density seeding toward high-throughput automated platforms that utilize micro-organoid configurations for early drug testing. Furthermore, the integration of real-time imaging and machine learning-assisted viability analysis allows for the detection of growth retardation long before traditional metabolic assays like CellTiter-Glo, potentially shaving days off the screening cycle. This temporal urgency is particularly acute in metastatic scenarios where multi-drug resistance, often mediated by the METTL3/YTHDF2/BCL2 axis or NAD^+^ metabolism reprogramming, necessitates rapid identification of salvage therapies or innovative targeted agents like CEACAM5-directed ADCs.

**FIGURE 4:**
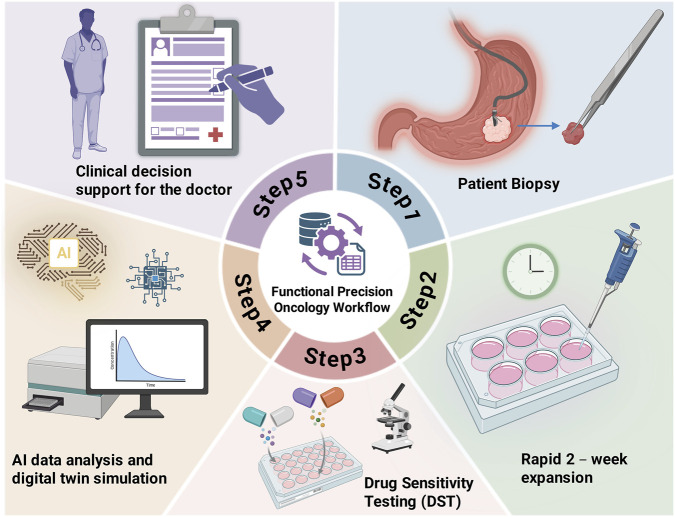
Integrated workflow of functional precision oncology (FPO) guided by PDCOs.

To achieve a higher level of “predictive fidelity,” the FPO workflow must evolve to incorporate the complex interactions of the TME without compromising the speed of decision-making (Ortiz Jordan et al., 2024). The next-generation of clinical decision tools involves “augmented organoids” that combine epithelial tumor cells with autologous CAFs and immune effectors (Sun et al., 2022; Xu et al., 2021). Innovative therapeutic platforms, such as NK cell-derived small extracellular vesicles engineered to target CLDN4, illustrate the potential for organoids to validate not only drug efficacy but also novel delivery mechanisms and radiosensitization strategies (Dong et al., 2025). Moreover, the application of spatial multi-omics and single-cell RNA sequencing to matched patient-organoid pairs allows clinicians to bridge the gap between regional tumor heterogeneity and functional readouts (Alonso et al., 2025). By utilizing molecular subtyping, including tsRNA-defined immune clusters, clinicians can prospectively stratify patients for drug sensitivity testing, thereby transforming GC-PDCOs from academic research tools into the “patient avatars” required for truly individualized precision management (Mircetic et al., 2023) (Figure 4).

### 5.3 Toxicity assessment and safety

The clinical utility of GC-PDCOs extends beyond predicting therapeutic efficacy to the critical domain of systemic toxicity assessment and safety profiling, addressing the narrow therapeutic window that often limits the administration of potent chemotherapeutic and targeted agents. Traditional preclinical toxicity models rely heavily on animal experiments, which frequently fail to account for human-specific physiological responses and the 3Rs principles of replacement, reduction, and refinement (Kim et al., 2026). By establishing organoid models from matched normal gastric mucosa and intestinal crypts, researchers can create a “toxicity avatar” to prospectively evaluate drug-induced gastrointestinal (GI) adverse events, such as nausea, vomiting, and diarrhea, which are primary drivers of dose reduction or treatment discontinuation in gastric cancer patients (Wu et al., 2024). Recent breakthroughs in organoid differentiation have enabled the construction of enterochromaffin (EC) cell-rich organoids that serve as high-sensitivity biosensors for drug-induced emesis (Hashimoto et al., 2025). These models can quantitatively measure the excessive release of serotonin in response to chemical stimuli, accurately distinguishing between high-emetic-risk agents like crizotinib and ceritinib and low-risk inhibitors like alectinib or entrectinib (Hashimoto et al., 2025; Salazar et al., 2023). Furthermore, the introduction of extrapolative parameters, such as the Dose/Fsk_40_ ratio, allows for the normalization of *in vitro* potency against clinical dosages, providing a robust framework for predicting individual patient sensitivity to emetic triggers and facilitating proactive dose adjustments.

The integration of GC-PDCOs into microfluidic multi-organ-on-a-chip (MOC) systems represents the next Frontier in assessing the systemic impact of oncological interventions (Liu et al., 2024). These advanced platforms facilitate the co-culture of gastric cancer organoids with liver, kidney, or heart modules, interconnected by biorealistic fluidic channels that mimic systemic circulation and drug metabolism (Zhou et al., 2024). Such systems are instrumental in parsing complex toxicity mechanisms, such as the UGT1A1-dependent enterotoxicity of irinotecan, where the metabolic conversion of the drug by liver modules dictates the local toxic exposure of matched intestinal organoids (Zhou et al., 2024). By replicating the physiological structures and functions of the human GI tract with high fidelity, including the assessment of epithelial barrier integrity via 90% accuracy in reproducing clinical diarrhea patterns, MOC platforms enable the identification of potential off-target effects and the evaluation of protective agents like MLN8054 or metformin to mitigate mucosal damage (Belair et al., 2020). This functional safety profiling allows for “precision dose-finding,” where the therapeutic regimen is tailored to achieve maximal oncogenic suppression while strictly remaining below the individual’s threshold for systemic toxicity, thereby optimizing both patient survival and quality of life.

## 6 Challenges and future perspectives

### 6.1 Modeling success rates and standardization bottlenecks

Despite the rapid progression of GC-PDCO technology, several fundamental bottlenecks remain that impede its integration into routine clinical practice, most notably the suboptimal modeling success rates and the lack of standardization across biobanking facilities. Current reports indicate an average success rate for GC-PDCO establishment that varies widely, often hovering around 48%–55% in many cohorts (Li et al., 2023). A primary determinant of this variable success is the inherent pathological subtype of the tumor. Signet ring cell carcinoma and other mucinous variants represent a significant hurdle; their submucosal infiltrative growth pattern and abundant intracellular mucus often result in insufficient yields of viable cancer cells during superficial mucosal biopsies, frequently leading to negative culture results (Roncati et al., 2018). Furthermore, tumors located in the gastric antrum exhibit significantly lower success rates (approximately 33%) compared to those in the gastric body (60%), a phenomenon likely attributed to the high prevalence of antral inflammation and edema, which disrupts tissue integrity and increases the risk of microbial contamination (Li et al., 2023). The presence of extensive necrotic tissue in advanced tumors further complicates establishment, as the initial sample size and the quality of highly active stem cell populations are critical prerequisites for successful 3D self-organization.

The absence of universally adopted standard operating procedures further exacerbates inter-laboratory variability. Establishing a rigorous standardized system for every step, from the depth and number of biopsies to the implementation of high-definition endoscopic staining, is essential to improve specimen quality (Huguet et al., 2022). Notably, recent studies have demonstrated that strict adherence to community-developed guidelines, such as JCR-level academic standards for gastrointestinal epithelial tissues, can elevate the success rate of gastrointestinal tumor organoid construction from roughly 50% to over 90% (Yang et al., 2024a). Beyond sampling, the technical proficiency of the operator is a non-negligible factor; senior endoscopists with more than 5 years of experience achieve significantly higher success rates compared to primary operators, highlighting the necessity for specialized training in organoid-focused biopsy techniques to ensure high-fidelity modeling (Shen et al., 2025). To address these persistent bottlenecks, we explicitly propose the establishment of a standardized Quality Control (QC) benchmarking system for “certified” clinical-grade organoids (Hong et al., 2025; Tan et al., 2024). This roadmap requires a tripartite validation framework: (1) Histopathological concordance, ensuring that PDCOs maintain key markers and glandular architecture of the parent tumor; (2) Genomic stability, verified through short tandem repeat (STR) profiling and the conservation of core driver mutations; and (3) Functional consistency, defined by standardized drug-response thresholds. Adhering to such community-developed benchmarks, combined with high-definition endoscopic staining to optimize specimen quality, is essential to elevate the success rates of mucinous and other challenging subtypes toward a 90% threshold (Yuan et al., 2026).

A secondary but equally critical bottleneck is the pervasive reliance on undefined ECM, primarily Matrigel. Although Matrigel provides a rich environment of basement membrane components, its composition is poorly defined and exhibits substantial batch-to-batch variability, which can introduce significant experimental bias and limit the reproducibility of functional assays (Cherne et al., 2021; Gebeyehu et al., 2021). Moreover, the murine origin of Matrigel raises animal welfare concerns and presents a regulatory barrier for future therapeutic applications in humans (Hassell et al., 1980). From a functional standpoint, the dense and complex structure of Matrigel has been shown to impede the migration of autologous immune cells, such as dendritic cells, thereby limiting the fidelity of co-culture models designed to study the tumor microenvironment (Hirth et al., 2024). Transitioning toward chemically defined, polysaccharide-based synthetic hydrogels, such as VitroGel ORGANOID-3, offers a promising solution by providing a controllable mechanical scaffold that supports both robust organoid growth and efficient immune cell motility (Cherne et al., 2021). Furthermore, integrating single-cell dissociation techniques during early passaging can help overcome the challenges of limited starting material and size heterogeneity, potentially shortening the drug screening cycle to a clinically actionable timeframe of 12–14 days (Gao et al., 2021). Addressing these standardization and matrix-related challenges is imperative for the transition of GC-PDCOs from academic research tools to validated platforms for precision oncology.

### 6.2 Technological breakthroughs: AI, 3D bioprinting and organ-on-a-chip

To address the existing translational bottlenecks, the next Frontier in gastric cancer research lies in the strategic convergence of bioengineering and computational intelligence, effectively transitioning from reductionist models to complex, “fit-for-purpose” ecosystems (Lim et al., 2026). Central to this evolution is the integration of 3D bioprinting, which allows for unprecedented spatial control over biochemical and mechanical signals within the ECM (Ju et al., 2025). Unlike conventional static cultures, bioprinted platforms utilize stomach-derived decellularized ECM scaffolds to recreate subtype-specific microenvironments (Kim et al., 2024). A landmark achievement in this domain is the incorporation of functional endothelial barriers within bioprinted gastric constructs, enabling the modeling of angiogenic responses and patient-specific drug sensitivities to agents like ramucirumab (Kim et al., 2024). This vascularized “stomach-like” model bridges the gap between simple epithelial clusters and the intricate human gastric architecture, fostering a more accurate representation of the tumor-interstitial interface (Liu et al., 2025b; Ferreira et al., 2023).

Complementing these structural advances, the implementation of microphysiological systems, or “stomach-on-a-chip” devices, introduces essential dynamic mechanical cues that are traditionally absent in organoid cultures (Hofer and Lutolf, 2021). By integrating organoids into microfluidic channels, researchers can simulate physiological peristalsis-like cyclic strain and fluid shear stress, which have been shown to significantly enhance epithelial maturation and promote the formation of a functional, in vivo-like mucus barrier (Shin et al., 2019). These dynamic forces are not merely ancillary; they modulate mechanotransduction pathways, such as the activation of PIEZO ion channels, which influence stem cell fate decisions and lineage differentiation (Baghdadi et al., 2024; Fang et al., 2025). Such engineering solutions provide a robust framework for investigating the complex interplay between mechanical tension and therapeutic resistance, particularly in the context of the stiffened ECM found in malignant gastric tissues.

The ultimate integration of these high-fidelity models with AI represents a paradigm shift toward truly predictive functional precision oncology (Lim et al., 2026). Specifically, AI directly addresses several persistent bottlenecks in PDO applications:

First, improving generation and culture success rates. To overcome low establishment rates and batch-to-batch variability, AI is revolutionizing organoid construction and culture optimization (Bai et al., 2024). AI algorithms can process data from sensors to monitor cellular behavior in real-time, allowing for dynamic, automated adjustments to culture conditions to optimize cell growth and health (Ahmed et al., 2025). This AI-enabled rapid screening of construction strategies ensures higher reproducibility and significantly improves the quality control of organoid fabrication (Maramraju et al., 2024).

Second, reducing cost and time. AI drastically reduces the time and financial burden associated with manual organoid maintenance and downstream analysis (Ahmed et al., 2025). In drug screening, deep learning models, such as convolutional neural networks (CNNs) and the OrganoID platform, automatically recognize, segment, and track single organoid dynamics pixel-by-pixel over time (Matthews et al., 2022). This automated 3D image processing eliminates labor-intensive manual profiling, accelerates high-throughput screening cycles, and effectively reduces reagent and labor costs (Oishi et al., 2024). For instance, automated image-based algorithms have already demonstrated utility in providing a rapid, non-destructive assessment of therapeutic impact such as cellular senescence (Yang et al., 2023).

Third, expanding the scope of applications. Beyond basic screening, AI fundamentally expands PDO utility by enabling the creation of “Artificial Intelligence Virtual Organoids” (AIVOs) or digital twins (Bai and Su, 2026). These silicon-based models fuse multimodal longitudinal data to emulate drug responses, allowing for high-throughput *in silico* experiments that predict optimal dosing without additional experimental burden (Bai and Su, 2026). Furthermore, machine learning facilitates the integration of PDO drug sensitivity profiles with multi-omics data to identify novel biomarkers and drive drug repurposing efforts (Maramraju et al., 2024; Radke et al., 2026). AI-driven digital pathology can even precisely distinguish tumor from normal PDOs in complex co-cultures (Doerfler et al., 2025), and provide highly sensitive quantitative assessments for environmental toxicity and therapeutic responses (Ge et al., 2025). As these models accumulate data, well-trained AI pipelines could eventually bypass the temporal constraints of individual organoid cultivation, rapidly predicting personalized treatment strategies from genomic data alone (Pauli et al., 2017).

### 6.3 Ethical, regulatory, and socioeconomic considerations

The clinical implementation of GC-PDCOs as functional decision-support tools introduces a complex landscape of ethical, regulatory, and socioeconomic challenges that must be addressed to ensure societal acceptance and sustainable integration into healthcare systems. At the core of these considerations is the evolving nature of informed consent, where the traditional “consent or anonymize” paradigm is increasingly rendered obsolete by the capacity for genomic re-identification through advanced big data analytics (Weidema et al., 2025; de Jongh et al., 2022). For gastric cancer patients, whose samples often contain sensitive genetic markers of hereditary risk, the scope of initial consent frequently fails to encompass the unforeseen longitudinal uses of their biomaterial in secondary research or commercial drug development. This has prompted a shift toward “dynamic consent” and “governance consent” models, which utilize digital platforms to facilitate ongoing two-way communication between researchers and donors, allowing patients to reshape their participation preferences as the technology evolves (de Jongh et al., 2022; Boers et al., 2015; de Groot et al., 2025). Such models are particularly relevant in the context of functional precision oncology, where the line between research and clinical care is blurred, necessitating a recalibration of the fiduciary duties of researchers to act in the best interests of the “patient-donor” (Lensink et al., 2020).

The ontological status of GC-PDCOs further complicates these ethical debates, as they are increasingly categorized not as mere biological “things” but as “hybrids” that possess both instrumental value as research tools and relational value as biological extensions of the donor (Ravn et al., 2023). Empirical studies indicate that donors often perceive an enduring personal connection to their organoids, viewing them as living fragments of themselves that require respectful stewardship (Haselager et al., 2020). This perception directly impacts the issue of ownership and commercialization. While commercial involvement is essential to provide the investment and infrastructure necessary for drug discovery, potential donors express significant reservations regarding “over-profit” and the commodification of their illness (Ravn et al., 2023). Many patients prioritize the “gift model” of altruistic donation but seek assurance that academic institutions, which they perceive as more trustworthy than private-sector entities, maintain leadership and ethical oversight in these collaborations. Furthermore, donors indicate that non-monetary appreciation, such as personalized thank-you notes or updates on scientific advancements enabled by their samples, can be more effective in sustaining participation than direct financial rewards (de Groot et al., 2025).

From a socioeconomic perspective, the distributive justice of organoid-guided therapies remains a primary concern. The average cost of precision therapies can exceed €75,000 per patient per year, creating a risk that the benefits of GC-PDCO technology will be accessible only to wealthy individuals or those in highly developed healthcare systems, thereby exacerbating existing global health inequities (Fleck, 2022). To achieve equitable implementation, pricing models must be adjusted to account for diverse socioeconomic backgrounds, and efforts must be made to ensure that biobank collections represent the full spectrum of genetic and ethnic diversity found in gastric cancer populations worldwide (Weidema et al., 2025). Failure to include underrepresented groups in the preclinical phase can result in “misguided evidence,” where the predictive models suffer from input bias and fail to deliver accurate responses for diverse populations (Dankwa-Mullan et al., 2025). Furthermore, ensuring that biobank collections reflect the full spectrum of genetic and ethnic diversity is a moral and scientific imperative to prevent misguided evidence in AI-driven models and to guarantee that the survival benefits of PDCO technology are accessible to patients across all socioeconomic backgrounds globally (Smith et al., 2025).

Regulatory frameworks are currently undergoing a period of rapid transition to accommodate these emerging technologies. The enactment of the FDA Modernization Act 2.0 in the United States and similar roadmaps in Europe reflect a significant regulatory receptivity toward “New Approach Methods”, including organoids and organ-on-chips, as alternatives to traditional animal testing (Han, 2023). However, for GC-PDCOs to serve as a true replacement for animal models in drug development, the field must overcome bottlenecks related to standardization and establish clear criteria for model validation and “predictive validity” (Leung et al., 2022). Furthermore, as these models gain complexity, issues of legal liability and accountability for diagnostic errors in n = 1 trials must be clarified. Ensuring a responsible future for GC-PDCO technology will ultimately depend on a “One Health” approach that integrates technical innovation with robust data governance, international regulatory harmonization, and a commitment to maintaining the social contract between science and society (Weidema et al., 2025).

## 7 Conclusion

The transformative trajectory of PDCOs has fundamentally redefined the landscape of gastric cancer research, elevating these models from specialized laboratory tools to a central pillar of precision oncology (Migliore et al., 2025; Wang et al., 2025a). GC-PDCOs provide an unparalleled biological interface that bridges the gap between static genomic data and the dynamic reality of a living tumor. Most significantly, their capacity to reconstruct the complex gastric TME, incorporating autologous immune cells, stromal architectures, and even the intragastric microecology, offers an irreplaceable platform for dissecting the mechanisms of immune evasion (Kan et al., 2025). By faithfully recapitulating the tripartite defenses of camouflage, coercion, and cytoprotection, organoid-based systems have become the gold standard for exploring the ecological complexity of gastric malignancies in a patient-specific context (Kan et al., 2025; Zhao et al., 2024).

Despite this progress, the path toward universal clinical implementation is marked by a critical need for standardization and technological synergy. Overcoming current bottlenecks, such as variable success rates in mucinous subtypes and the reliance on animal-derived matrices, remains a translational imperative. However, the ongoing integration of PDCOs with spatial multi-omics, 3D bioprinting, and artificial intelligence is poised to catalyze a paradigm shift in therapeutic decision-making (Alonso et al., 2025; Lim et al., 2026). AI-augmented workflows can now analyze high-throughput functional screening data with unprecedented speed, potentially delivering actionable clinical insights within the narrow temporal window required for advanced gastric cancer care (Baisiwala et al., 2026; Yu, 2025). This synergistic convergence promises to resolve the “scalability-fidelity” trade-off, transforming PDCOs into high-velocity engines for drug discovery and safety-first toxicity profiling (Schmäche et al., 2024).

Looking forward, we are entering a new era of “patient avatars” where the clinical management of gastric cancer will undergo a conceptual leap from biomarker-based selection to functional validation (Liu et al., 2025e; Ren et al., 2022). In this emerging landscape, therapeutic regimens will no longer be dictated solely by a “lookup table” of genetic mutations, but by the empirical *ex vivo* response of a patient’s own tumor cells. By integrating these high-fidelity models into routine clinical workflows, we can achieve truly individualized oncology, sparing patients from ineffective toxicities while identifying the most potent combinations of chemotherapy, targeted agents, and immunotherapies (Liu et al., 2024). Ultimately, the maturation of GC-PDCO technology represents a strategic roadmap toward a future where every gastric cancer patient receives a treatment plan as unique as their own biology, realizing the full promise of functional precision medicine (Skubleny et al., 2025).

## Glossary

GC: Gastric cancer
ICIs: Immune checkpoint inhibitors
PDCOs: Patient-derived cancer organoids
3D: Three-dimensional
2D: Two-dimensional
GC-PDCOs: Gastric cancer organoids
ALI: Air-liquid interface
TME: Tumor microenvironment
PDXs: Patient-derived xenografts
ITH: Intratumoral heterogeneity
scRNA-seq: Single-cell RNA sequencing
GIM: Gastric intestinal metaplasia
1C: One-carbon
CIN: Chromosomal instability
MSI: Microsatellite instability
GS: Genomically stable
DGC: Diffuse-type gastric cancer
EMT: Epithelial-to-mesenchymal transition
AQP5: Aquaporin-5
CSCs: Cancer stem cells
ECM: Extracellular matrix
PD-L1: Programmed death-ligand 1
ROS: Reactive oxygen species
GSH: Glutathione
CAFs: Cancer-associated fibroblasts
MSCs: Mesenchymal stem cells
5-FU: 5-fluorouracil
GCOs: Gastric cancer organoids
H. pylori: *Helicobacter pylori*
CDK1: Cyclin-dependent kinase 1
TLRs: Toll-like receptors
EBV: Epstein-barr virus
DST: Drug sensitivity testing
PI3: Peptidase inhibitor 3
ADC: Antibody-drug conjugate
T-DXd: Trastuzumab deruxtecan
GSDMB: Gasdermin B
CLDN18.2: Claudin 18.2
TKIs: Tyrosine kinase inhibitors
CAR: Chimeric antigen receptor
IFN-γ: Interferon-gamma
tsRNAs: Transfer RNA-derived small RNAs
PDT: Photodynamic therapy
DCA: Deoxycholic acid
IM: Intestinal metaplasia
PFS: Progression-free survival
OS: Overall survival
EOX: Epirubicin, oxaliplatin, and capecitabine
SEM: Stem-like/EMT/mesenchymal
CAV1: Caveolin-1
FPO: Functional precision oncology
GI: Gastrointestinal
EC: Enterochromaffin
MOC: Multi-organ-on-a-chip
AI: Artificial intelligence
